# Role of Heme-Oxygenase-1 in Biology of Cardiomyocytes Derived from Human Induced Pluripotent Stem Cells

**DOI:** 10.3390/cells10030522

**Published:** 2021-03-01

**Authors:** Mateusz Jeż, Alicja Martyniak, Kalina Andrysiak, Olga Mucha, Krzysztof Szade, Alan Kania, Łukasz Chrobok, Katarzyna Palus-Chramiec, Anna M. Sanetra, Marian H. Lewandowski, Ewelina Pośpiech, Jacek Stępniewski, Józef Dulak

**Affiliations:** 1 Department of Medical Biotechnology, Faculty of Biochemistry, Biophysics and Biotechnology, Jagiellonian University, 30-387 Krakow, Poland; mateusz.jez@doctoral.uj.edu.pl (M.J.); alicja.martyniak@doctoral.uj.edu.pl (A.M.); kalina.andrysiak@doctoral.uj.edu.pl (K.A.); olga.mucha@doctoral.uj.edu.pl (O.M.); krzysztof.szade@uj.edu.pl (K.S.); 2 Department of Neurophysiology and Chronobiology, Institute of Zoology and Biomedical Research, Jagiellonian University, 30-387 Krakow, Poland; alan.kania@uj.edu.pl (A.K.); lukasz.chrobok@uj.edu.pl (Ł.C.); k.palus@uj.edu.pl (K.P.-C.); anna.sanetra@doctoral.uj.edu.pl (A.M.S.); marian.lewandowski@uj.edu.pl (M.H.L.); 3 Human Genome Variation Research Group, Malopolska Centre of Biotechnology, Jagiellonian University, 30-387 Krakow, Poland; ewelina.pospiech@uj.edu.pl

**Keywords:** hiPSC-CMs, HO-1, CRISPR/Cas9, electrophysiology, hypertrophy, IGF2

## Abstract

Heme oxygenase-1 (HO-1, encoded by *HMOX1*) is a cytoprotective enzyme degrading heme into CO, Fe^2+^, and biliverdin. HO-1 was demonstrated to affect cardiac differentiation of murine pluripotent stem cells (PSCs), regulate the metabolism of murine adult cardiomyocytes, and influence regeneration of infarcted myocardium in mice. However, the enzyme’s effect on human cardiogenesis and human cardiomyocytes’ electromechanical properties has not been described so far. Thus, this study aimed to investigate the role of HO-1 in the differentiation of human induced pluripotent stem cells (hiPSCs) into hiPSC-derived cardiomyocytes (hiPSC-CMs). hiPSCs were generated from human fibroblasts and peripheral blood mononuclear cells using Sendai vectors and subjected to CRISPR/Cas9-mediated *HMOX1* knock-out. After confirming lack of HO-1 expression on the protein level, isogenic control and HO-1-deficient hiPSCs were differentiated into hiPSC-CMs. No differences in differentiation efficiency and hiPSC-CMs metabolism were observed in both cell types. The global transcriptomic analysis revealed, on the other hand, alterations in electrophysiological pathways in hiPSC-CMs devoid of HO-1, which also demonstrated increased size. Functional consequences in changes in expression of ion channels genes were then confirmed by patch-clamp analysis. To the best of our knowledge, this is the first report demonstrating the link between HO-1 and electrophysiology in human cardiomyocytes.

## 1. Introduction

The leading cause of death in developed countries, despite significant advances in pharmacotherapy and cardiac surgery, are cardiovascular diseases, including ischemic heart diseases (IHD) [1]. Myocardial infarction (MI) leads to ischemia and hypoxia of the heart muscle, resulting in loss of up to one billion cardiomyocytes (approximately 25% of all cardiomyocytes found in the left ventricle) [2]. Due to the adult human heart’s negligible regenerative capabilities, a scar is formed in place of lost cardiomyocytes. A non-contractile collagen tissue further weakens electromechanical properties of a damaged hearts, which may lead to arrhythmias, severe failure, and eventually, death [3]. Despite significant advances in cardiology, organ transplantation is the only solution for many patients with chronic heart failure. However, the possibility of transplantations is limited by an insufficient number of donors. A promising alternative to heart transplantations may be a rapidly growing field of personalized and regenerative medicine.

Human induced pluripotent stem cells (hiPSCs) can be generated from easily accessible somatic cells, such as fibroblasts, peripheral blood mononuclear cells, or even urine-derived epithelial cells from a particular patient [4,5,6]. hiPSCs can be then differentiated into virtually all somatic lineages found in the adult organism, including cardiomyocytes (hiPSC-CMs) [7,8], thus giving invaluable opportunity for investigation of their regenerative potential for the human heart. However, further studies are needed to better understand hiPSCs cardiac differentiation, particularly considering the immature state of hiPSC-CMs [9]. Despite not fully understood biology, hiPSC-CMs are already widely used in academia and industry for drug screening, where they are successfully replacing murine and other animal models [10].

One of the enzymes crucial for cardiac development is heme oxygenase-1 (HO-1; encoded by *HMOX1*). HO-1 is a cytoprotective enzyme degrading heme into carbon monoxide (CO), ferrous ions (Fe^2+^), and biliverdin. Products of enzymatic activity of HO-1 have a plethora of biological effects such as modulating angiogenesis, inflammation and differentiation of stem cells [11,12,13]. Piantadosi et al. [14] have proven HO-1 to be crucial for mitochondrial biogenesis in murine cardiomyocytes, which might be of great importance considering the metabolic immaturity of iPSC-CMs. Our recent studies have also proven the HO-1 importance in the murine cardiomyocytes’ biology [15,16]. We showed that HO-1-lacking murine iPSC formed significantly less beating clusters during spontaneous in vitro differentiation via embryoid bodies, indicating an essential role of HO-1 in cardiomyogenesis [15]. Additionally, previously we have shown adverse late left ventricle remodelling in HO-1-deficient mice upon induction of MI [16].

Here we aim to validate our and other’s studies regarding HO-1 in murine cardiomyocyte biology in the human model of the hiPSC-derived cardiomyocytes.

## 2. Materials and Methods

### 2.1. Differentiation of hiPSCs into Cardiomyocytes

Verified HPSI1013i-kuxp_1 hiPSC (named hiPSC.2) has been purchased from The Human Induced Pluripotent Stem Cells Initiative’s collection (www.hipsci.org, accessed on 9 January 2017). hiPSC.1 was reprogrammed from commercially available BJ fibroblasts (ATCC, CRL-2522), and hiPSC.3 were reprogrammed from PBMCs isolated from a healthy donor (approval of the Institutional Review Board and Bioethical Committee and with informed consent, in accordance with the Declaration of Helsinki—nr. of approval 122.6120.303.2016). Both hiPSC.1 and hiPSC.3 were reprogrammed using non-integrating Sendai vectors (Cytotune-iPS 2.0 Sendai Reprogramming kit, cat. #A16517; ThermoFisher Scientific, Waltham, MA, USA) according to the manufacturer’s protocol. The characteristics of all donors and list of the experiments they were used for are provided in Table 1. The pluripotency of used hiPSC lines was confirmed as described by us previously before using the cells in further experiments [15,17]. Shortly, obtained hiPSCs were stained for pluripotency markers: NANOG, OCT-3/4, SSEA-4, TRA-1-60, and TRA-1-81. Then hiPSCs were spontaneously differentiated via embryoid bodies (EBs) in the Essential 6 (E6) medium (ThermoFisher Scientific). After 2 weeks, cells were stained for markers of all three germ layers: mesoderm (Vimentin and α-smooth muscle actin (α-SMA)); endoderm (GATA4 and alpha-fetoprotein (AFP)); and ectoderm (neurofilament heavy chain (NFH)).

Verified hiPSCs were cultured as described previously [18]. hiPSCs were differentiated to cardiomyocytes utilising the GiWi protocol, described by Lian et al. [8]. Shortly, CHIR99021 and IWR-1 (both Sigma-Aldrich, Saint Louis, MO, USA) were used as small molecules regulating the WNT pathway. Cells were subjected to metabolic selection from day 10 until day 16 [19], with an additional reseeding step on day 13.

Differentiated hiPSC-CMs were phenotyped as described previously [20]. Shortly, on day 20–24 of differentiation, cardiomyocytes were harvested, fixed, and stained for TNNT2 (Cardiac Troponin T Monoclonal Antibody, 1:1000, clone 13–11; ThermoFisher Scientific). The percentage of TNNT2^+^ cells was determined using LSRFortessa flow cytometry analyzer (BD Biosciences, Franklin Lakes, NJ, USA) and BD Software.

### 2.2. Karyotyping

Karyotype analysis was performed by Kariogen laboratory (Krakow, Poland) (G-banding method).

### 2.3. Generation of HO-1 KO hiPSCs

HO-1 knock-out (HO-1 KO) hiPSCs were generated using the CRISPR/Cas9 gene-editing method. Method and sgRNAs were described elsewhere [21] (oligonucleotides used for cloning of *HMOX1*-targeting portion of sgRNA into plasmid are listed in the Supplementary Table S1). To minimize the possible off-target effect on the experiments, three different sgRNAs were tested: sgRNA1, sgRNA2, and sgRNA3. Due to the lack of cleavage of DNA in sgRNA3, only the first two were used for further experiments. After nucleofection with either empty Cas9 plasmid (control) or specific sgRNAs (sgRNA1 and sgRNA2) targeting *HMOX1* using Human stem cell nucleofector kit 1 (Lonza, Basel, Switzerland), hiPSCs were subjected to antibiotic selection with puromycin (0.5–0.7 µg/mL) for 24 h. After 3–4 days, DNA was isolated from the cells using Genomic Mini kit (A&A Biotechnology, Gdynia, Poland) according to the manufacturer’s protocol and Surveyor assay (home-made at the Department of Cellular Biochemistry, Faculty of Biochemistry, Biophysics and Biotechnology, Jagiellonian University, Krakow, Poland) was performed, to confirm the activity of Cas9 and sgRNAs. hiPSCs were then reseeded in low density to obtain single-cell derived clones. Single-cell derived clones were subjected to the HO-1 level evaluation using the Western blot technique.

### 2.4. Western Blot

Protein was isolated and Western blot was performed as described previously [15]. Membranes were blotted for HO-1 (#ADI-SPA-894-F, 1:200, Enzo Life Sciences, Farmingdale, NY, USA) and α-tubulin as a reference (#T5168, 1:1000, Sigma-Aldrich, Saint Louis, MO, USA). 

### 2.5. Immunofluorescence Analysis

hiPSCs or hiPSC-CMs were reseeded 3 days before staining (except spontaneous differentiation via EBs). The staining procedure was performed, as described previously [22]. Antibodies are listed in Table 2.

### 2.6. RNA Isolation and qRT-PCR

RNA isolation, reverse transcription, and qRT-PCR were conducted as described previously [15]. Specific primers for each gene are listed in Table 3.

### 2.7. Oxygen Consumption Rate (OCR)—Seahorse Assay

Oxygen consumption rate (OCR) was measured using Seahorse Bioscience XFe96 Analyzer (Agilent Technologies, Santa Clara, CA, USA). Then, 10,000 hiPSC-CMs were seeded into Seahorse XFe96-well plates. After 3 days, hiPSC-CMs were stimulated with 10 µM CoPPIX for 48 and 72 h. On the day of the experiment, a medium was switched to a low-buffered assay medium (8.3 g/L DMEM, 2 mM L-Glutamine, and 0.5% phenol red; pH 7.4—all from Sigma-Aldrich, Saint Louis, MO, USA) and incubated at 37 °C, 20% O_2_, without CO_2_ for 1 h. OCR was assessed after sequential injections of oligomycin (1.5 µg/mL), FCCP (0.8 µM), and Rotenon+Antymycin A (both 1 µM (all from Sigma-Aldrich, Saint Louis, MO, USA)) were performed. Based on OCR, the following parameters were calculated: basal respiration, ATP production, maximal respiration, non-mitochondrial respiration, spare respiratory capacity, and proton leak.

### 2.8. Mitochondrial Membrane Activity—TMRM Assay

The activity of mitochondrial membranes was measured using the TMRM compound (ThermoFisher Scientific), which binds to active mitochondrial membranes. On day 20–24 of differentiation, 50,000 hiPSC-CMs were seeded on 24 well plates. After 3 days, hiPSC-CMs were treated with 10 µM CoPP or SnPP for 24, 48 and 72 h. Next, cells were harvested using TrypLE (ThermoFisher Scientific). 50,000 hiPSC-CMs were resuspended in 500 µL of RPMI + B27 + TMRM (20 nM), and incubated at 37 °C for 30 min. Then, cells were centrifuged at 200× *g* for 5 min and resuspended in RPMI+B27 w/o TMRM. Median TMRM fluorescence was measured using LSRFortessa flow cytometry analyzer (BD Biosciences, Franklin Lakes, NJ, USA) and BD Software (gating strategy is presented in Supplementary Figure S1).

### 2.9. Transcriptome Analysis

RNA from hiPSCs and hiPSC-CMs (2 × 10^6^ cells per sample) was isolated using mirVana™ miRNA Isolation Kit (ThermoFisher Scientific), according to vendors’ protocol. Targeted, whole transcriptome profiling was performed using next-generation sequencing and a highly multiplexed amplification method provided by Ion AmpliSeqTM technology and Ion ProtonTM machine (ThermoFisher Scientific). Libraries for eight undifferentiated hiPSC and eight differentiated hiPSC-CM samples were prepared using Ion AmpliSeq™ Transcriptome Human Gene Expression Panel, which precisely defines the expression of over 20,000 human RefSeq genes in a single assay. Before library preparation, RNA samples were evaluated for their integrity, and their concentrations were measured using Agilent 2100 Bioanalyzer with RNA 6000 Nano Kit (Agilent, Santa Clara, CA, USA). Subsequently, two pools of libraries for eight undifferentiated hiPSC and eight differentiated hiPSC-CM samples were prepared according to the manufacturer’s protocol. Then, libraries were sequenced on Ion Proton Sequencer with Ion PI Hi-Q Sequencing 200 Kit and two Ion PI Chips v3. The primary bioinformatic analyses were carried out on Torrent Suite Server v5.12.1. Reads were aligned to the hg19 AmpliSeq Transcriptome ERCC v1 reference and counted with Torrent Coverage Analysis Plugin. Gene expression data were normalized, and differential gene expression analysis was carried out using the DESeq2 package (with default parameters) implemented in R version 3.3.3 software [23]. *p*-values for differentially expressed genes were corrected for multiple comparisons using the Benjamini-Hochberg approach. Data were deposited in the BioProject database (ID 687272).

### 2.10. Patch-Clamp Analysis

For the patch-clamp analysis hiPSC-CMs were seeded at low density (10,000 cells per 10 mm glass coverslip). Then, 3 days after seeding, hiPSC-CMs were measured (Figure 7) or treated with 10 µM CoPP for 72 h and then measured (Figures 8 and 9). hiPSC-CMs electrical activity was measured using a whole-cell current clamp with borosilicate electrodes (7–9 MΩ) containing 125 mM K-gluconate, 20 mM KCl, 5 mM NaCl, and 10 mM HEPES. Cells were continuously superfused with extracellular solution containing: 140 mM NaCl, 5.4 mM KCl, 1.8 mM CaCl_2_ × 2H_2_O, 1 mM MgCl_2_ × 6H_2_O, 5.5 mM glucose, and 5 mM HEPES (pH = 7.4), at a temp. of 37 °C. Signal was amplified using SC 05LC amplifier (NPI). Data were low pass filtered at 2 kHz and digitized at 20 kHz. Data was acquired using Signal software (Cambridge Electronic Design Inc., Cambridge, UK).

All experiments were conducted in current-clamp mode. For action potential (AP) measurements, membrane potential was manually adjusted to −60 mV with the negative current injection and held steady throughout the experiment (one exception was made for experiments shown in Figure 7, where the membrane potential was kept on the native value). To evoke single AP, short (2 ms), rectangular positive current injections (from 150 pA to 210 pA) were applied every 1 s, to ensure the pacing of 1 Hz. Ten consecutive APs were averaged, and the shape of the waveform was analyzed in Signal software (CED). The AP kinetics were characterized by measuring (1) upstroke velocity and (2) time from the AP peak to three discrete values of 20, 50, and 90% repolarization; termed as action potential duration (APD) 20, 50, and 90. Additionally, the maximal depolarization was calculated as ‘action potential peak’ while the through hyperpolarization following the spike as an ‘after hyperpolarization’ value (AHP).

### 2.11. Cell Size Measurement

To measure the cell size, hiPSC-CMs were reseeded at low density (10,000 cells per cm^2^). Then, 3 days after reseeding, cells were fixed and stained for TNNT2 (as described elsewhere [22]), to distinguish cardiomyocytes from non-cardiomyocytes. Immunofluorescence pictures were taken, and cell size was measured using ImageJ software. 

### 2.12. Statistical Analysis

Data are presented as mean ± SD of 3 independent experiments (differentiation batches) unless stated otherwise. To analyze statistical significance, t-test for two groups comparisons or one-way ANOVA followed by Dunnett’s test for multiple comparisons were used. Statistical analyses were performed using GraphPad Prism software. *p* < 0.05 was considered as statistically significant. DEseq2 package was used for the bioinformatical analysis of RNA-seq results [23].

## 3. Results

### 3.1. Generation of HO-1 KO hiPSCs Lines

To verify the role of HO-1 in the process of hiPSCs cardiac differentiation, we have used three hiPSC lines, originating from different donors. Firstly, the pluripotency of all hiPSCs lines employed in this study was checked. hiPSC.1 expressed pluripotency markers: NANOG, OCT4, SSEA4, TRA-1-60, TRA-1-81 (Supplementary Figure S2A), and spontaneously differentiated in vitro via EBs into cells originating from three germ layers (mesoderm, endoderm, and ectoderm—Supplementary Figure S2B). Karyotype of all hiPSCs was examined (Supplementary Figure S3) Pluripotent properties of hiPSC.2 and hiPSC.3 were characterized in our previous studies [17,18] (respectively). 

**Figure 1:**
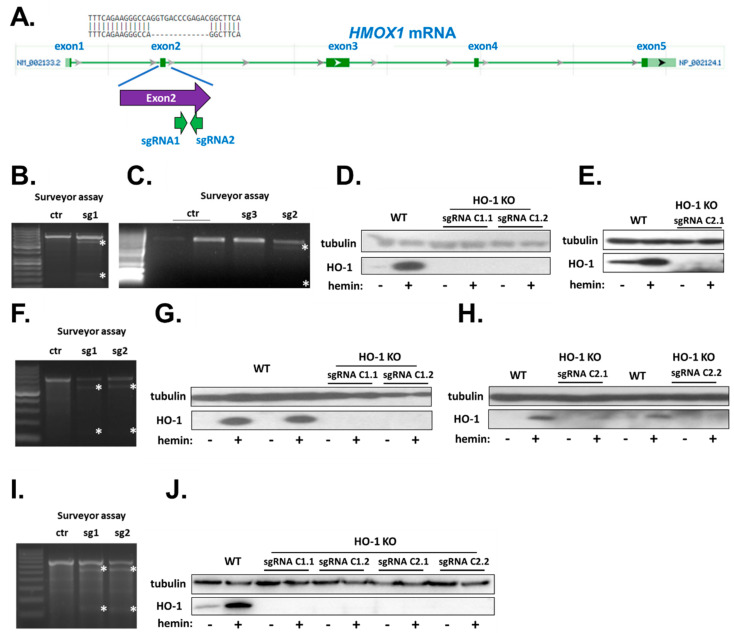
Generation of HO-1 KO hiPSC lines. (**A**) Schematic representation of *HMOX1* mRNA and sgRNAs-targeted sites with a representative sequencing result of HO-1 KO line. Adapted from ncbi.nlm.nih.gov. Surveyor assay of hiPSCs after nucleofection with specific sgRNAs: (**B**,**C**) hiPSC.1, (**F**) hiPSC.2 and (**I**) hiPSC.3. Asterisks indicate products of cleavage of heteroduplexes. Functional confirmation of HO-1 knockdown on protein level after hemin stimulation in (**D**,**E**) hiPSC.1, (**G**,**H**) hiPSC.2, and (**J**) hiPSC.3. WT—isogenic control hiPSCs.

HO-1 KO hiPSC clones were generated from the characterized lines through the nucleofection with a plasmid encoding Cas9 and designed sgRNAs targeting *HMOX1* exon 2, described elsewhere [21], schematically illustrated in Figure 1A. After nucleofection with sgRNAs (and empty Cas9 plasmid without sgRNA sequences as control) and selection with puromycin (0.5–0.7 µg/mL, 24 h), surveyor assay was performed to confirm the presence of mutations in *HMOX1* exon 2 in all hiPSC lines (Figure 1B,C,F,I). Then, single-cell derived clones were stimulated with hemin, a known potent activator of *HMOX1* transcription and subjected to protein isolation and Western blot assay to functionally confirm lack of HO-1 expression (Figure 1D,E,G,H,J). Selected clones were expanded and used in further experiments.

### 3.2. Verifying Level of GATA6 and Pluripotency of HO-1 KO hiPSC Clones

**Figure 2:**
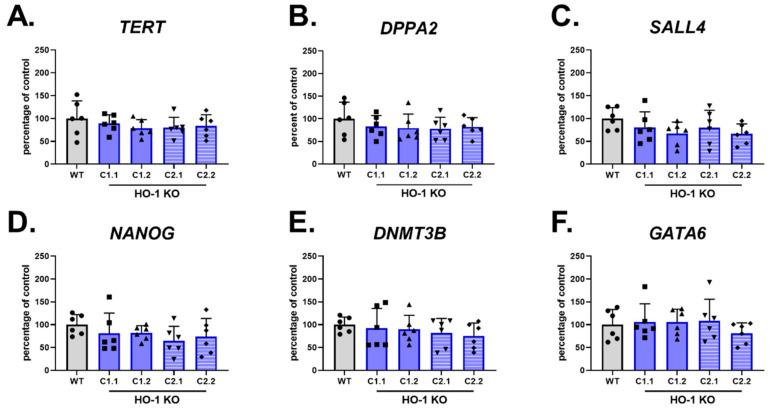
Lack of HO-1 does not influence the level of *GATA6* and pluripotency markers. qRT-PCR analysis of (**A**–**E**) pluripotency markers and (**F**) *GATA6* in WT and HO-1 KO hiPSC.3. Expression was normalized to *EEF-2* levels. Bars represent mean ± SD of N = 3 experiments. Dots, squares and triangles represent each replicate for corresponding groups, one-way ANOVA test.

HO-1 KO hiPSC.3 clones, originating from sgRNA1 (C1 clones) and sgRNA2 (C2 clones) were harvested 2 days after passaging for RNA isolation. qRT-PCR analysis of pluripotency markers: *NANOG*, *TERT*, *DPPA2*, *SALL4*, and *DNMT3B* (Figure 2A,E, respectively) did not reveal any changes in tested genes in comparison to the control counterpart (nucleofected with empty Cas9 plasmid), demonstrating that lack of HO-1 does not affect hiPSCs function. The level of *GATA6*, a predictor of hiPSC cardiomyocyte differentiation capability [24], was also not changed (Figure 2F).

### 3.3. HO-1 Does Not Influence Cardiomyocyte Differentiation Efficiency

To assess whether HO-1 might affect the efficiency of hiPSCs cardiac differentiation, we have performed direct differentiation of control (WT) and HO-1 KO hiPSC.1 clones into cardiomyocytes, without the step of metabolic selection. In the first experiment, two HO-1 KO clones had slightly higher efficiency measured as the percentage of cardiac troponin T (TNNT2)-positive cell. In contrast, other HO-1 KO clones had lower efficiency than WT cells (Supplementary Figure S4A). However, the percentage of TNNT2-positive cells in the second experiment was much lower in both WT and HO-1 KO hiPSC clones (Supplementary Figure S4B). These observations indicated high variances in directed differentiation efficiency between experiments. Thus, it was not possible to assess the role of HO-1 in the development of human cardiomyocytes using this method.

**Figure 3:**
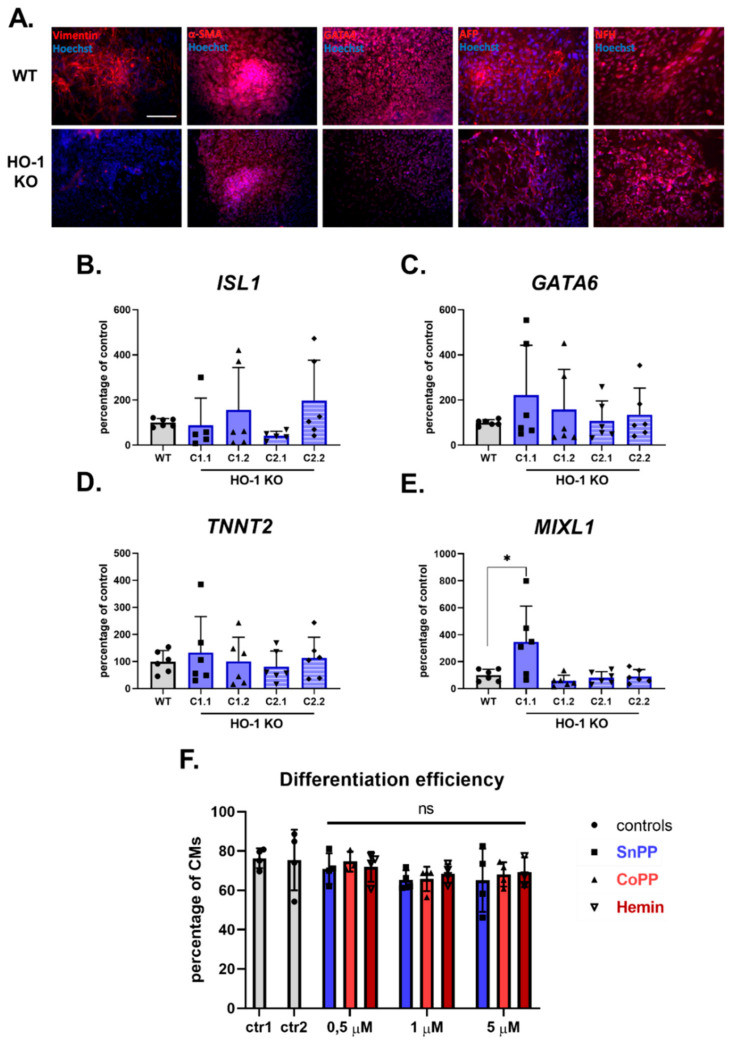
HO-1 does not influence the efficiency of hiPSCs.2 differentiation to cardiomyocytes. (**A**) Immunofluorescence analysis of markers of three germ-layers (Vimentin, α-SMA, GATA4, AFP, and NFH) in spontaneously differentiated WT (upper panel) and HO-1 KO (bottom panel) hiPSC.3 via embryoid bodies. Bar indicates 100 µm. qRT-PCR analysis of expression of cardiac mesoderm markers: (**B**) *ISL1*, (**C**) *GATA6*, (**D**) *TNNT2* and (**E**) *MIXL1.* Expression was normalized to *EEF-2* levels. (**F**) Flow cytometric analysis of direct cardiomyocyte differentiation efficiency (based on TNNT2 expression) of WT hiPSC.2 treated with tin protoporphyrin IX (SnPP), cobalt protoporphyrin IX (CoPP) and hemin. Ctr1-DMSO control for SnPP and CoPP, ctr2-2 µM NaOH control for hemin. Bars represent mean ± SD of N = 3 experiments. Dots, squares and triangles represent each replicate for corresponding groups. * *p* < 0.05, one-way ANOVA test.

Therefore, in the next step, we have employed the technique of spontaneous in vitro differentiation of WT and HO-1 KO hiPSC clones via embryoid bodies. Of note, EBs were spontaneously differentiated in DMEM medium supplemented with 20% FBS, instead of standard E6 medium, which contains insulin, reported to inhibit cardiac differentiation and favor neuronal development [25]. Both WT, HO-1 KO C1.1 (Figure 3A), and HO-1 KO C1.2, C2.1, and C2.2 (data not shown) differentiated into cells originating from all three germ layers. Interestingly, one of the mesoderm markers—vimentin—was not detectable in all HO-1 KO clones. qRT-PCR analysis of cardiac mesoderm markers in spontaneously differentiated cells, on the other hand, revealed no differences in *ISL1*, *GATA6*, and *TNNT2*(Figure 3B–D, respectively) expression in HO-1 KO EBs compared to WT control. The level of another cardiac mesoderm—*MIXL1* was significantly upregulated in only one out of four tested HO-1 KO clones (Figure 3E). However, the analysis of the other clones makes the interaction of HO-1 and *MIXL1* questionable.

In the last approach to verify the role of HO-1 in cardiomyocyte development, we applied pharmacological modulators of HO-1 activity: inhibitor—tin protoporphyrin IX (SnPP), and activators—cobalt protoporphyrin IX (CoPP) and hemin [26]. WT hiPSC.2 were directly differentiated into cardiomyocytes in the presence of 0.5; 1, and 5 µM SnPP, CoPP, and hemin (10 µM concentration of all tested compounds was toxic to the cells in the long term—data not shown). Flow cytometric analysis of the TNNT2-positive cells revealed that pharmacological modulation of HO-1 activity in WT hiPSCs did not influence cardiomyocyte differentiation efficiency (Figure 3F). Representative flow cytometry images are shown in Supplementary Figure S5.

### 3.4. HO-1 Induction by CoPP Stimulation Does Not Influence the Metabolism of hiPSC-CMs

Suliman et al. [27] have previously demonstrated that the HO-1/CO system enhances mitochondria’s maturation in cardiomyocytes derived from murine embryonic stem cells. Therefore, in the next step, we aimed to verify these results in the human model.

**Figure 4:**
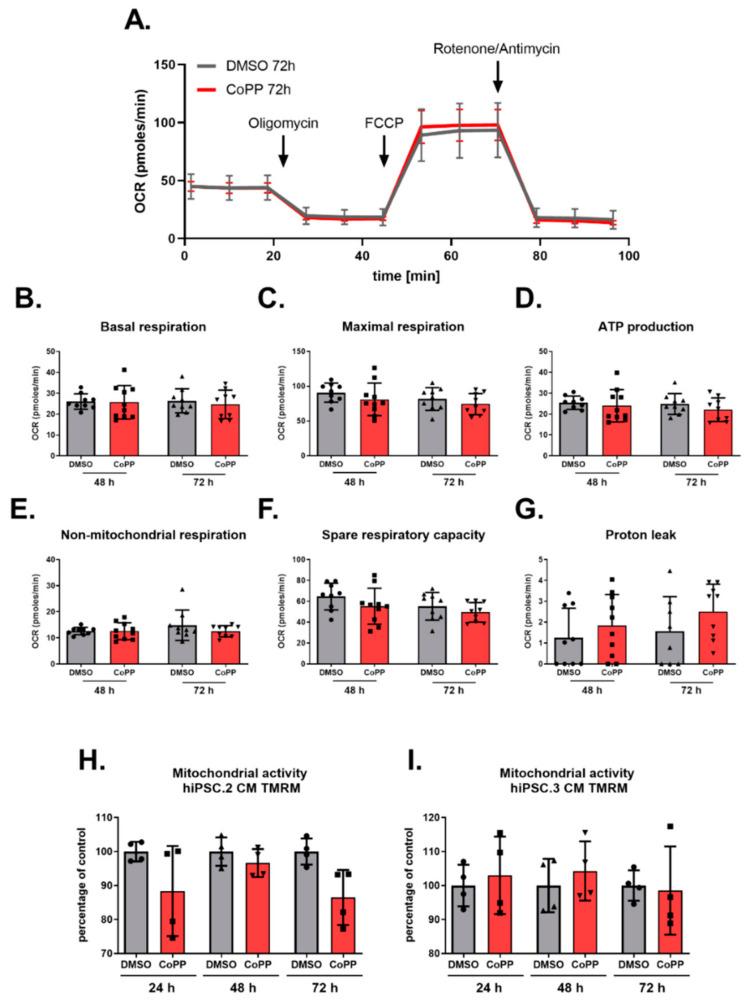
Stimulation of HO-1 expression by cobalt protoporphyrin IX (CoPP) does not influence the metabolic activity of hiPSC-CMs. Seahorse analysis of hiPSC.3-CMs treated with CoPP (**A**–**D**). (**A**) Graphical illustration of Seahorse results. (**B**) Basal respiration, (**C**) maximal respiration and (**D**) ATP production. Tetramethylrhodamine (TMRM) assay of (**E**) hiPSC.2-CMs and (**F**) hiPSC.3-CMs treated with CoPP. Bars represent median ± SD of N = 2 experiments. Dots, squares and triangles represent each replicate for corresponding groups, one-way ANOVA test.

Analysis of the oxygen consumption rate (OCR) of hiPSC.3-CMs treated with CoPP did not reveal any changes in the metabolism (Figure 4A). 48 and 72 h treatment with 10 µM CoPP did not influence the level of any assessed parameters: basal respiration, maximal respiration, ATP production, non-mitochondrial respiration, spare respiratory capacity, and proton leak (Figure 4B–G, respectively).

In the complementary assay, the activity of mitochondrial membranes was measured using tetramethylrhodamine (TMRM), a fluorescent dye sequestering in active mitochondria membranes. hiPSC.2 and hiPSC.3-CMs were treated with 10 µM CoPP for 24, 48, and 72 h or SnPP for 48 and 72 h. Further, flow cytometric analysis did not reveal any fluorescence intensity changes, suggesting that activation of HO-1 by CoPP (or inhibition by SnPP—Supplementary Figure S6) did not increase mitochondrial activity (Figure 4H,I). Representative flow cytometry images are shown in Supplementary Figure S7.

### 3.5. Transcriptome Analysis of WT and HO-1 KO hiPSCs and hiPSC-CMs

As the knockdown of HO-1 did not influence the pluripotency of hiPSCs, differentiation efficiency, nor mitochondrial activity, we decided to gain more in-depth insight into the potential role of HO-1 on hiPSCs and hiPSC-CMs biology. Accordingly, we performed RNA-seq analysis of undifferentiated and differentiated WT and HO-1 KO hiPSC clones. 

**Figure 5:**
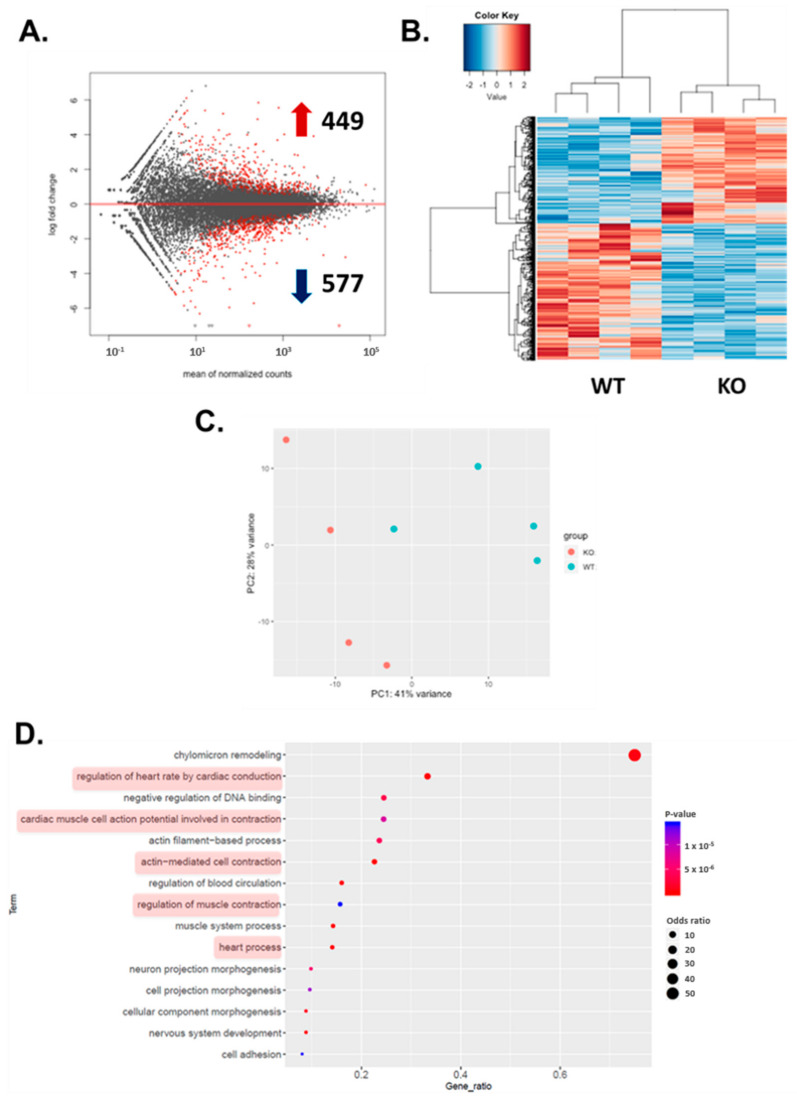
Transcriptomic analysis of hiPSC.3-CMs WT and HO-1 KO. (**A**) Plot with differentially expressed genes. 449 were upregulated and 577 downregulated. (**B**) Hierarchical clustering of differentially expressed genes. (**C**) Principal component analysis. (**D**) Analysis of gene ontology (GO) terms.

Comparison of undifferentiated WT and HO-1 KO hiPSCs revealed no differences in gene expression profile between both genotypes (Supplementary Figure S8) whereas cardiac differentiation, as expected, imposed substantial transcriptomic changes in the analyzed cells (hiPSCs vs hiPSC-CMs, Supplementary Figure S8). Notably, more than 1000 differentially expressed genes (DEG) distinguished the WT and HO-1 KO hiPSC-CMs, among which 449 genes were upregulated, and 577 were downregulated (Figure 5A). In parallel, hierarchical clustering and principal component analysis (PCA) clearly separated WT, and HO-1-deficient hiPSC-CMs (Figure 5B,C). Gene ontology analysis of DEG revealed that five out of the top 15 altered biological processes were related to cardiomyocytes’ electrophysiology (marked in red).

### 3.6. Altered Ion Channel Expression in HO-1 KO hiPSC-CMs

**Figure 6:**
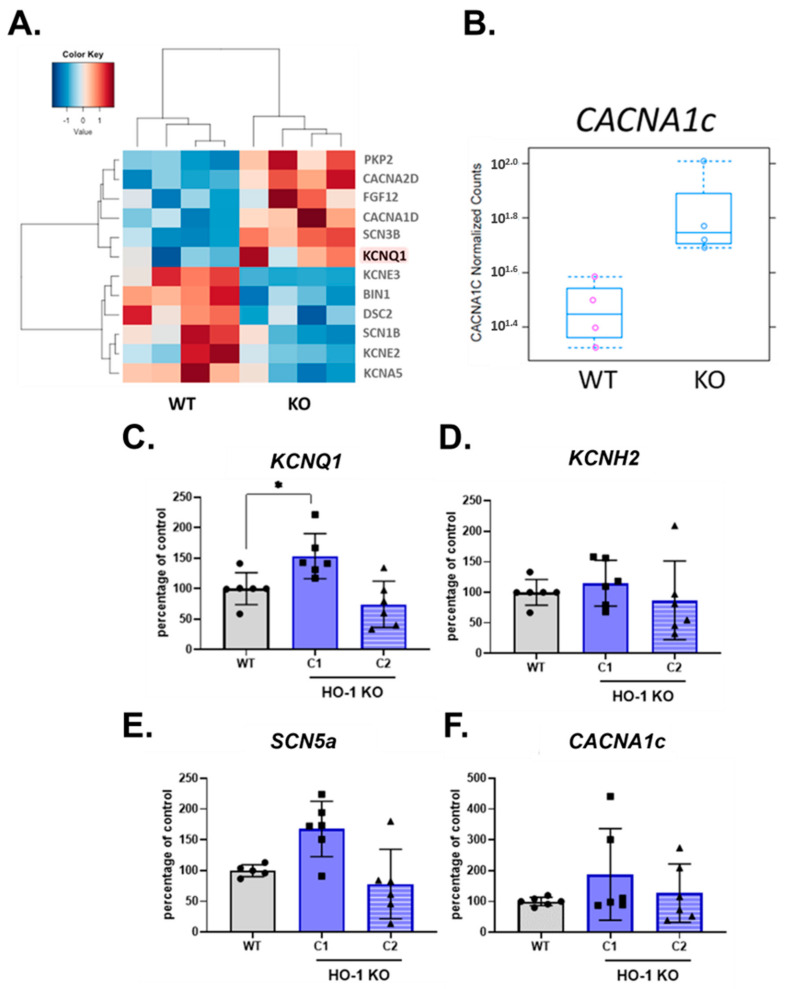
Expression of ion channels in WT and HO-1 KO hiPSC.3-CMs. (**A**) RNA-seq-GO term: cardiac muscle cell potential involved in contraction. (**B**) RNA-seq: normalized counts for *CACNA1c* gene. qRT-PCR analysis of (**C**) *KCNQ1*, (**D**) *KCNH2*, (**E**) *SCN5a*, and (**F**) *CACNA1c*. Expression was normalized to *TNNT2* levels. Bars represent mean ± SD of N = 3 experiments. Dots, squares and triangles represent each replicate for corresponding groups. * *p* < 0.05, one-way ANOVA test.

Transcriptomic analysis of WT and HO-1 KO hiPSC-CMs revealed changes vastly in processes related to electrophysiological properties (Figure 5D). A closer insight into GO term ‘cardiac muscle cell potential involved in contraction’ indicated for increased expression of potassium channel *KCNQ1* and lower level of other potassium channels: *KCNE2, KCNE3, KCNA5*, compared to WT counterparts. The level of calcium channels transcripts was increased (*CACNA1d* and *CACNA2d*) (Figure 6A). However, among upregulated calcium channels not assigned to the GO term mentioned above, was *CACNA1c* (Figure 6B). The qRT-PCR analysis confirmed upregulation of the KCNQ1 gene in HO-1 KO hiPSC-CMs (Figure 6C), however only in case of one out of two tested HO-1 KO clones. Based on qRT-PCR, we did not observe the changed expression of other genes encoding ion channels crucial for hiPSC-CMs biology: potassium *KCNH2*, sodium *SCN5a*, and calcium *CACNA1c*.

### 3.7. Shortened Action Potential Duration in HO-1 KO hiPSC-CMs

**Figure 7:**
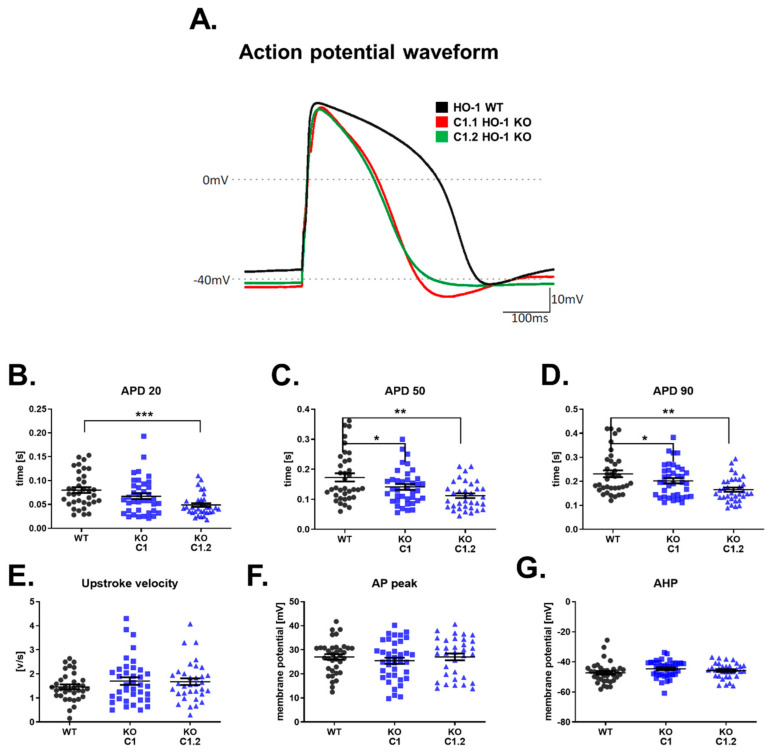
Electrophysiological properties of WT and HO-1 KO hiPSC.1-CMs. (**A**) Representative AP waveforms for WT and HO-1 KO hiPSC.1-CMs. Action potential duration (APD) at (**B**) 20%, (**C**) 50%, and (**D**) 90% repolarization. (**E**) Upstroke velocity. (**F**) AP peak, and (**G**) AHP. N = 32–38 cells. Dots, squares and triangles represent each measurement for corresponding groups. * *p* < 0.05, ** *p* < 0.01, *** *p* < 0.005, one-way ANOVA test.

Analysis of RNA-seq results suggested changes in electrophysiological properties (Figure 5D). However, more in-depth insight into one of the GO terms (cardiac muscle cell potential involved in contraction) revealed an inconsistent direction in the changes. The qRT-PCRs analysis did not clarify these observations. Therefore, we performed a functional measurement of electrophysiological properties of HO-1 KO hiPSC-CMs. The whole-cell patch-clamp analysis shown reduced APD manifested as faster repolarization in both HO-1 KO hiPSC-CMs clones. (Figure 7A–D). The upstroke velocity, AP peak, and AHP parameters remained unchanged (Figure 7E–G).

### 3.8. Effect of CoPP on Electrophysiological Properties of WT and HO-1 KO hiPSC-CMs

As we found a link between lack of HO-1 and upregulation of potassium channel expression, KCNQ1, and what was subsequently reflected in the AP’s decreased duration, we asked whether pharmacological stimulation of HO-1 with CoPP will also impact the electrophysiological properties of hiPSC-CMs.

**Figure 8:**
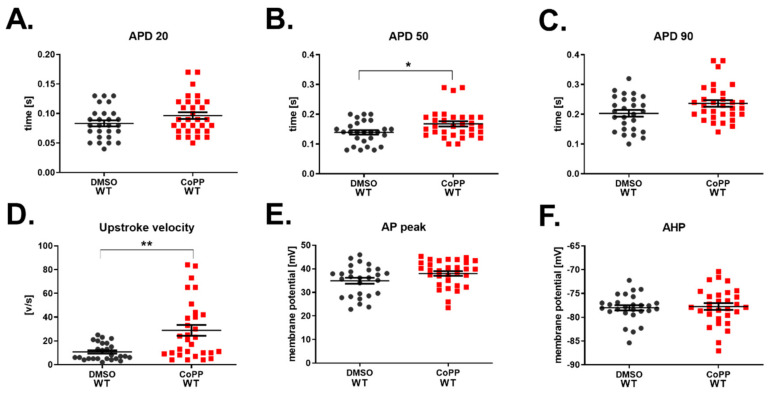
Electrophysiological properties of WT hiPSC.2-CMs stimulated with CoPP. Action potential (AP) duration at (**A**) 20%, (**B**) 50%, and (**C**) 90% repolarization. (**D**) Upstroke velocity, (**E**) AP peak, and (**F**) AHP. N = 27–34 cells. Each dot and square represents one measurement for corresponding groups. * *p* < 0.05, ** *p* < 0.01, *t*-test.

Indeed, WT hiPSC.2 CMs treated with 10 µM CoPP had increased APD50 (Figure 8B). However, the difference in APD20 and APD90 did not reach the significance threshold (Figure 8A,B). Interestingly, upstroke velocity was potently increased in hiPSC-CMs treated with CoPP (Figure 8D). Values of AP peak and AHP remained unchanged (Figure 8E,F).

**Figure 9:**
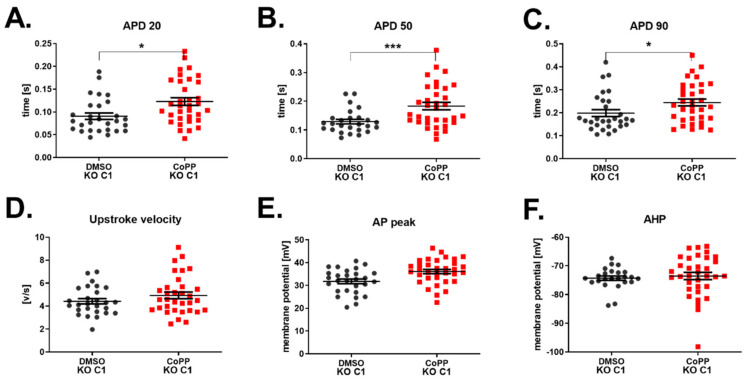
Electrophysiological properties of HO-1 KO hiPSC.2-CMs stimulated with CoPP. APD at (**A**) 20%, (**B**) 50%, and (**C**) 90% repolarization. (**D**) Upstroke velocity, (**E**) AP peak, and (**F**) AHP. N = 27–34 measurements. Each dot and square represents one measurement for corresponding groups. * *p* < 0.05, *** *p* < 0.001, *t*-test.

Unexpectedly, HO-1 KO hiPSC-CMs treated with 10 µM CoPP were also characterized by a similar increase in APD (Figure 9A–C) as WT hiPSC-CMs (Figure 8B). That indicates that CoPP may act on potassium channels, but its mechanism is not dependent on HO-1. However, the upstroke velocity, which depends on the activity of sodium channels, remained unaffected. As we observed an increase of upstroke velocity in WT hiPSC-CMs treated with CoPP (Figure 8D), but not in HO-1 KO hiPSC-CMs (Figure 9D), we might conclude that this is an HO-1-dependent mechanism of CoPP action on sodium conductance.

Western blot analysis confirmed at the protein level CoPP-mediated HO-1 upregulation in WT hiPSC-CMs and lack of HO-1 upregulation in HO-1 KO hiPSC-CMs (Supplementary Figure S9A,B, respectively).

### 3.9. Regeneration Pathway and Cell Size of HO-1 KO hiPSC-CMs

**Figure 10:**
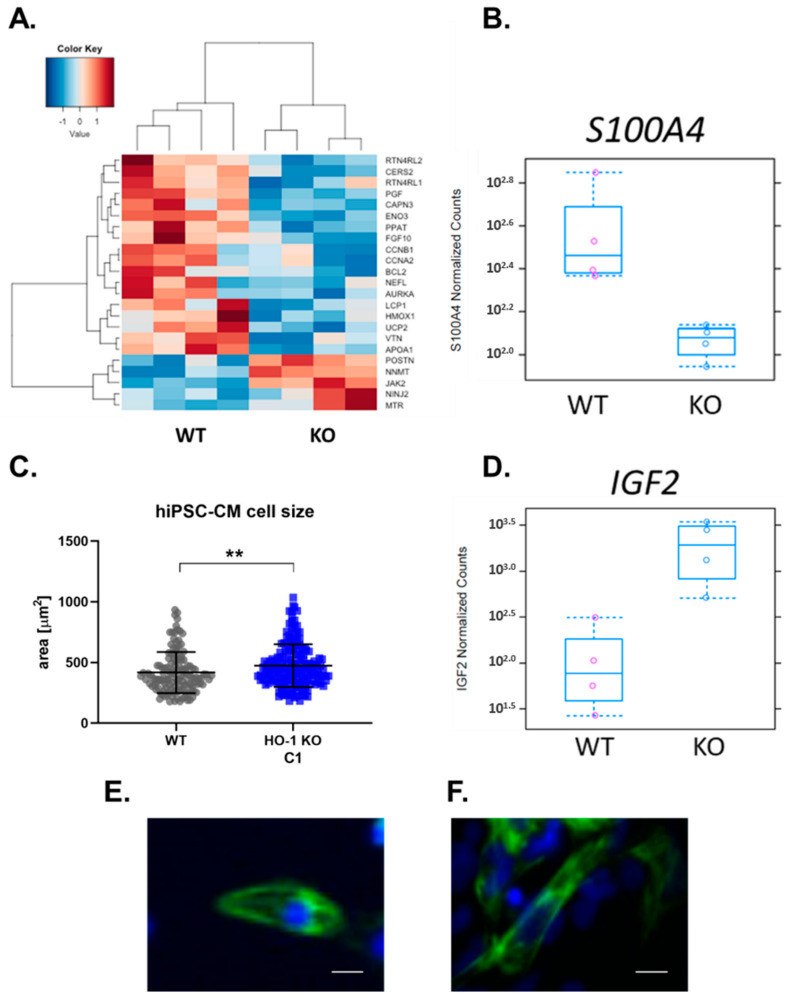
Regeneration pathway and cell size of WT and HO-1 KO hiPSC.3-CMs. (**A**) RNA seq: GO term-Regeneration, (**B**) RNA-seq: normalized counts for *S100A4* gene. (**C**) The cell size of 133 WT and 184 HO-1 KO hiPSC-CM. Dots and squares represent each measurement for corresponding groups. ** *p* < 0.01, *t*-test. (**D**) RNA-seq: normalized counts for *IGF2* gene. Representative picture of (**E**) WT, and (**F**) HO-1 KO hiPSC-CM. Bar indicates 5 µm.

Among over 1000 genes changed in HO-1 KO hiPSC-CMs, were those involved in regeneration. The observed pattern suggests the decreased regenerative potential of HO-1 KO hiPSC-CMs (Figure 10A). What is important is, expression of a critical factor involved in the recovery of the infarcted heart-*S100A4*-was also remarkably lowered, based on RNA-seq results (Figure 10B). Additionally, on average, hiPSC-CM lacking HO-1 were 13.4% (±4.8%) bigger than hiPSC-CM WT (Figure 10C); this observation is also supported by increased expression of *IGF2*, a key factor involved in cardiac hypertrophy (Figure 10D).

## 4. Discussion

We report that modulation of HO-1 does not affect pluripotency of human iPSCs. Accordingly, in HO-1 KO hiPSCs clones level of pluripotency markers remained unchanged, and they could spontaneously differentiate to cells originating from all three germ layers. Neither genetically engineered knockdown of HO-1 nor pharmacological modulation of HO-1 did not influence hiPSC differentiation efficiency to cardiomyocytes. However, one of the mesoderm markers—vimentin, was undetectable in HO-1 KO embryoid bodies. Of note, HO-1 was shown to regulate vimentin [28]. Furthermore, CoPP-mediated HO-1 induction in hiPSC-CMs did not enhance their metabolism, based on two independent experimental approaches. Next, based on transcriptome and qRT-PCR analyses of ion channels expression, we have assessed the electrophysiological properties of WT and HO-1 KO hiPSC-CMs. Of note, the importance of HO-1 in the electrophysiology of hiPSC-CMs was confirmed using the patch-clamp method. However, more detailed studies of this phenomenon were not possible due to the HO-1-independent action of CoPP on the electrophysiology of CMs, which has not been reported so far.

Nevertheless, RNA-seq analysis demonstrated more than 1000 differentially expressed genes between control and HO-1-deficient cardiomyocytes, belonging to many important biological processes, particularly involved in the electrophysiological activity of the heart.

### 4.1. HO-1 Does Not Influence the Stemness of hiPSCs

Here, we reported a similar level of pluripotency markers in both WT and HO-1 KO hiPSCs. These observations align with our and other studies regarding the HO-1 and pluripotency in murine stem cells [15,29]. In both studies, lack of HO-1 in murine embryonic stem cells (ESCs) and murine iPSCs did not affect their stemness, as the pluripotency markers were at the same level both in WT and HO-1 KO cells. Lin et al. [29], on the other hand, linked genetic and pharmacological downregulation of cytoprotective HO-1 with a more rapid decline in pluripotency markers (Oct4, SSEA-1, alkaline-phosphatase activity) in stem cells subjected to spontaneous differentiation. Increased intracellular ROS, which might be the effects of lack of HO-1 activity, has been linked with facilitated withdrawal from quiescence and faster differentiation of pluripotent and multipotent stem cells [30,31]. Therefore, modulation of HO-1 at the early stages of in vitro differentiation of pluripotent stem cells might be of great interest. However, it should be stressed that differentiation from the stem cell to the end-stage somatic cells is a long process involving a plethora of molecular pathways, differently regulated at various stages of differentiation. Therefore, the incautious modulation of HO-1 in stem cells might have unexpected in vivo effects. As shown by our team, the lack of HO-1 in the bone marrow niche led to the extensive exhaustion of hematopoietic stem cells, most probably due to the aforementioned facilitation of differentiation in HO-1 KO stem cells [32]. On the other hand, upregulation of HO-1 in progenitor cells might have an even more deleterious effect in vivo. As also shown by our team, overexpression of HO-1 in muscle progenitor cells led to their uncontrolled proliferation and formation of tumor-like structure in vivo [33], which might be of relevance for rhabdomyosarcoma development [34].

### 4.2. HO-1 Does Not Influence the Differentiation Efficiency of hiPSCs into Cardiomyocytes

Basing on the described role of HO-1 in cardiac differentiation of mouse PSCs [15,27], one could expect a clear pattern in the efficiency of cardiomyocyte differentiation from hiPSCs with modulated HO-1 activity. 

However, CRISPR/Cas9-mediated knockdown of HO-1 did not affect cardiac mesoderm markers’ expression in spontaneously differentiated EBs. Similarly, pharmacological stimulation or inhibition of HO-1 during direct hiPSCs differentiation into hiPSC-CMs did not affect the efficiency of this process. These findings were additionally confirmed by the lack of change in the *GATA6* gene in HO-1 KO hiPSCs, which was shown to be the predictor of PSC differentiation capability toward cardiomyocytes [24].

Directed cardiomyocyte differentiation is a highly complex process, composed of sequential steps of mesoendoderm induction (by activation of the Wnt pathway), which then goes through cardiac mesoderm (induced by subsequent inhibition of the Wnt pathway), cardiac progenitors and finally give rise to spontaneously contracting cardiomyocytes [7]. As molecular pathways are oppositely regulated at different stages of this process, it may suggest that HO-1 should be manipulated in a time-spatial manner. Indeed, a similar idea has been reviewed by Wei and Cong [35], where authors, basing on numerous studies (and sometimes seemingly contradictory) regarding ROS and cardiac differentiation, concluded that at the early stage of differentiation, elevated ROS is required, but for proper further cardiac differentiation drop in ROS is essential.

Therefore, in our study, we might conclude that lack of any effect of HO-1 modulation on the efficiency of differentiation of hiPSCs into cardiomyocytes might be because of constant either upregulation or downregulation of HO-1 throughout the whole differentiation process. The initial positive effect of HO-1 modulation could be blurred due to too long exposure to given stimuli.

### 4.3. HO-1 Does Not Influence the Metabolic Activity of hiPSC-CMs

Cardiomyocytes derived from hiPSCs are ready for measurement within approximately 20 days [7], whereas the human heart reaches maturity in the early twenties [36]. Such a short time of in vitro differentiation and lack of interaction with other cell types found in the developing heart do not allow for the full maturation of hiPSC-CMs. Transcriptomic analysis performed by others revealed that hiPSC-CMs resemble human fetal cardiomyocytes in the first trimester of development [9]. Among many properties of hiPSC-CMs, which are immature compared to adult cardiomyocytes, underdeveloped mitochondria are one of the main hurdles in the maturation of hiPSC-CMs [37]. 

Suliman et al. [27] investigated the role of HO-1/CO system in the process of metabolic maturation of energy-demanding cardiomyocytes derived from spontaneously differentiated murine ESCs. The authors reported that the CO, a product of the enzymatic activity of HO-1, increased mitochondrial biogenesis, and their structural network. 

Our study aimed to confirm the role of HO-1 in the maturation of mitochondria in cardiomyocytes in human cells. Nevertheless, hiPSC-CMs treated with CoPP, the inductor of HO-1 activity, did not show any signs of mitochondrial maturation, as indirectly measured by oxygen consumption rate (Seahorse analysis) and directly by assessing the activity of mitochondrial membranes (TMRM analysis).

The fact that findings regarding the murine heart’s metabolism are not reflected in human cardiomyocytes is not surprising. Firstly, due to anatomical differences, murine hearts are characterized by an almost ten times higher beating rate than humans [38]. Therefore it might be expected that murine cardiomyocytes, to meet their large energy demand, will be characterized by differently regulated metabolism, compared to their human counterparts, which are beating much slower.

### 4.4. HO-1 Alters Electrophysiological Properties of hiPSC-CMs

The hypothesis concerning the potential role of HO-1 in the electrophysiological activity of hiPSC-CMs is based on the well-known effect of CO on the activity of ion channels (reviewed in [39]). 

The transcriptomic analysis of WT and HO-1 KO hiPSC-CMs demonstrated that five out of the top 15 changed biological processes were related to cardiac conductivity. Detailed analysis of the GO term ‘cardiac muscle cell potential involved in contraction’ suggested increased expression of potassium channel KCNQ1. Increased influx of potassium ions during AP results in faster repolarization, manifested by shortened AP (role of ion channels in electrophysiology of hiPSC-CMs reviewed in: [40]). This finding was confirmed by patch-clamp analysis, as shortened APD characterized the HO-1 KO hiPSC-CMs. Decreased expression of other potassium channels (KCNE2, KCNE3, KCNA5), which activity also leads to decreased APD, might be explained as a compensatory effect. Increased calcium channels expression also supports the hypothesis about the compensatory effect, as their activity has the opposite, to potassium channels, effect on APD [40]. Altered activity of discussed ion channels is characteristic for atrial fibrillation [40].

Further, detailed studies with CoPP, the inductor of HO-1 activity, failed to provide clear answers, as CoPP exhibited HO-1-independent mechanism of action, which was noticed in HO-1 KO hiPSC-CMs. Of note, HO-1-independent CoPP activity was already reported in other studies; however, none of them was related to cardiomyocytes and electrophysiology [41,42,43,44]. 

Despite the unexpected effect of CoPP, increased upstroke velocity after the CoPP treatment of hiPSC-CMs WT seemed dependent on HO-1 upregulation. That could be potentially of great interest, as increased upstroke velocity was reported in the case of maturating hiPSC-CMs [45]. However, the other hallmark of maturation is the increased expression of ion channels [46]. In the case of HO-1 KO hiPSC-CMs, we have observed an inconsistent pattern of ion channels expression. Considering that potassium ions’ equilibrium is disrupted in the heart after MI [47], the disrupted potassium ion currents in HO-1 KO hiPSC-CMs should be regarded as a pathological effect.

The hypothesis about pathologically altered electrophysiology of hiPSC-CMs lacking HO-1 is supported by changes in genes involved in regeneration (RNA-seq results). Of importance, expression of *S100A4*, a gene which was recently shown to be crucial for heart regeneration after MI [48,49], was also downregulated in HO-1 KO hiPSC-CMs. Of note, changes in genes involved in regeneration may reflect, reported by Tomczyk et al., adverse late left ventricle remodelling in HO-1-deficient mice upon induction of MI [16]. Pathological effect of HO-1-deficiency is additionally supported by, reported here, increased expression of IGF2-a key regulator of cardiac hypertrophy [50], which was followed by increased HO-1 KO hiPSC-CMs size. Cardiomyocyte hypertrophy was also reported in HO-1-deficient hearts characterized by impaired regeneration [16]. 

## 5. Conclusions

In the present study, we show that the HO-1 does not impact the efficiency of hiPSCs differentiation into cardiomyocytes, nor the mitochondrial activity of hiPSC-CMs. However, the results indicate pathologically altered electrophysiology of HO-1 KO hiPSC-CMs. Our data is potentially of great importance, due to the polymorphism of the *HMOX1* promoter, which results in variations of HO-1 level in the population and impacts susceptibility for coronary artery disease [51]. Also, commonly used drugs were shown to modulate the HO-1 [22,52], which in consequence may affect the electrophysiological activity of the heart. However, to better understand this phenomenon, more detailed studies on male and female hiPSC-CMs are needed as female hiPSC-CMs were recently shown to be more prone to electrophysiological abnormalities [53]. 

## Figures and Tables

**Table 1:** Characteristics of the human induced pluripotent stem cell (hiPSC) donors.

hiPSC	Sex	Tissue Type	Age (Years)	Metabolism	RNA-seq	Patch-Clamp
*hiPSC.1*	male	fibroblasts	newborn			x
*hiPSC.2*	male	fibroblasts	25–29	x		x
*hiPSC.3*	male	PBMC	55	x	x	

**Table 2:** List of antibodies.

Antibody	Dilution	Vendor (cat. nr.)
Pluripotency markers		
Oct-3/4	1:200	Santa Cruz (sc8628)
NANOG	1:100	Santa Cruz (sc33759)
SSEA4	1:100	Millipore (90231)
TRA 1-61	1:100	Millipore (90232)
TRA 1-81	1:100	Millipore (90233)
Markers of three germ layers		
Vimentin	1:250	Abcam (ab92547)
α-SMA	1:200	Abcam (ab5694)
GATA4	1:200	Santa Cruz (sc25310)
AFP	1:200	Santa Cruz (sc-8108)
NFH	1:200	Abcam (ab8135)
Cardiac specific marker		
TNNT2	1:200	ThermoFisher Scientific (MA5-12960)

**Table 3:** List of specific primers used for qRT-PCR.

Gene	Primer 1	Primer 2
*CACNA1c*	CAGAGGCTACGATTTGAGGA	GCTTCACAAAGAGGTCGTGT
*DNMT3B*	GGAGAAAGCTAGGGTGCGAG	AATTCCCTACTGCCTGCAGGA
*DPPA2*	CCGTCCCCGCAATCTCCTTCCATC	ATGATGCCAACATGGCTCCCGGTG
*EEF2*	TCAGCACACTGGATAGAGG	GACATCACCAAGGGTGTGCA
*GATA6*	TCCCCCACAACACAACCTAC	TGTAGAGCCCATCTTGACCC
*ISL1*	TGATGAAGCAACTCCAGCAG	GGACTGGCTACCATGCTGTT
*KCNH2*	AATCGCCTTCTACCGGAAAG	CACCATGTCCTTCTCCATCAC
*KCNQ1*	TCTGTCTTTGCCATCTCCTTC	CCTCCATGCGGTCTGAATG
*MIXL1*	GGTACCCCGACATCCACTT	GAGACTTGGCACGCCTGT
*NANOG*	GAAGACAAGGTCCCGGTCAA	ACCATTGCTATTCTTCGGCCA
*SALL4*	TGTGGCGGAGAGGGCAAATA	GTGGCTTCATCCTCACTCGC
*SCN5A*	GAGCTCTGTCACGATTTGAGG	GAAGATGAGGCAGACGAGGA
*TNNT2*	ATCCAGAACGCCCAGACAGA	GCTGCTTGAACTTCTCCTGC

## Data Availability

RNA-seq results were deposited at BioProjects database (ID 687272).

## Supplementary Materials

The following are available online at https://www.mdpi.com/2073-4409/10/3/522/s1, Table S1. List of primers used for cloning of HMOX1-targeting portion of sgRNA into plasmid, Figure S1. Gating strategy of hiPSC-CMs stained with TMRM, Figure S2. Characterization of hiPSC.1. (A.) Immunofluorescence analysis of pluripotency markers (NANOG, OCT4, SSEA4, TRA-1-61, and TRA-1-80). (B.) Immunofluorescence analysis of markers of three germ-layers of spontaneously differentiated embryoid bodies (GATA4, Vimentin, α-smooth muscle actin, alpha-fetoprotein, and neurofilament heavy chain. Bar indicates 100 µm, Figure S3. Karyotype analysis of (A) hiPSC.1 and (B.) hiPSC.2. Arrows indicating t(6;15)(p21.2;q15) reciprocal translocation. Figure S4. Flow cytometric analysis of cardiac differentiation efficiency of WT and HO-1 KO hiPSC.1, based on TNNT2 expression. (A.) Frist differentiation, (B.) Second differentiation. Two replicates in each differentiation. Figure S5. Differentiation efficiency. Representative flow cytometry images. Figure S6. Tetramethylrhodamine (TMRM) assay of hiPSC-CMs treated with SnPP. Bars represent median ± SD of N=1 experiment (2 replicates). Figure S7. TMRM assay of hiPSC.3-CMs. Representative flow cytometry images. Figure S8. Transcriptomic analysis of undifferentiated WT and HO-1 KO hiPSCs and hiPSC-CMs. Hierarchical clustering of differentially expressed genes in undifferentiated (left side) and differentiated cells (right side). Figure S9. Western blot: confirmation of HO-1 induction (or lack of) by CoPP on the protein level in (A.) WT hiPSC.2-CMs and (B.) HO-1 KO hiPSC.2-CMs.
