# Supplementary material for: Role of Heme-Oxygenase-1 in Biology of Cardiomyocytes Derived from Human Induced Pluripotent Stem Cells
Source: Cells. 2021 Mar 1;10(3):522. doi: 10.3390/cells10030522 (PMC8000937; doi:10.3390/cells10030522)
Supplement: Supplementary file 1 [file cells-10-00522-s001.pdf]

cells

MDPI

Supplementary information

Role of heme-oxygenase-1 in biology of cardiomyocytes derived from human induced pluripotent stem cells

Mateusz Jeż[1], Alicja Martyniak[1], Kalina Andrysiak[1], Olga Mucha[1], Krzysztof Szade[1], Alan Kania[2], Łukasz Chrobok[2], Katarzyna Palus-Chramiec[2], Anna M. Sanetra[2], Marian H. Lewandowski[2], Ewelina Pośpiech[3], Jacek Stępniewski[1*#], Józef Dulak[1*#]

Table 1. List of primers used for cloning of *HMOX1*-targeting portion of sgRNA into plasmid.

Gene	Primer 1	Primer 2
sgRNA1	CACCGTGAAGCCGTCTCGGGTCACC	AAACGGTGACCCGAGACGGCTTCAC
sgRNA2	CACCGCCTCCTTGGTGGCCTCCTTC	AAACGAAGGAGGCCACCAAGGAGGC

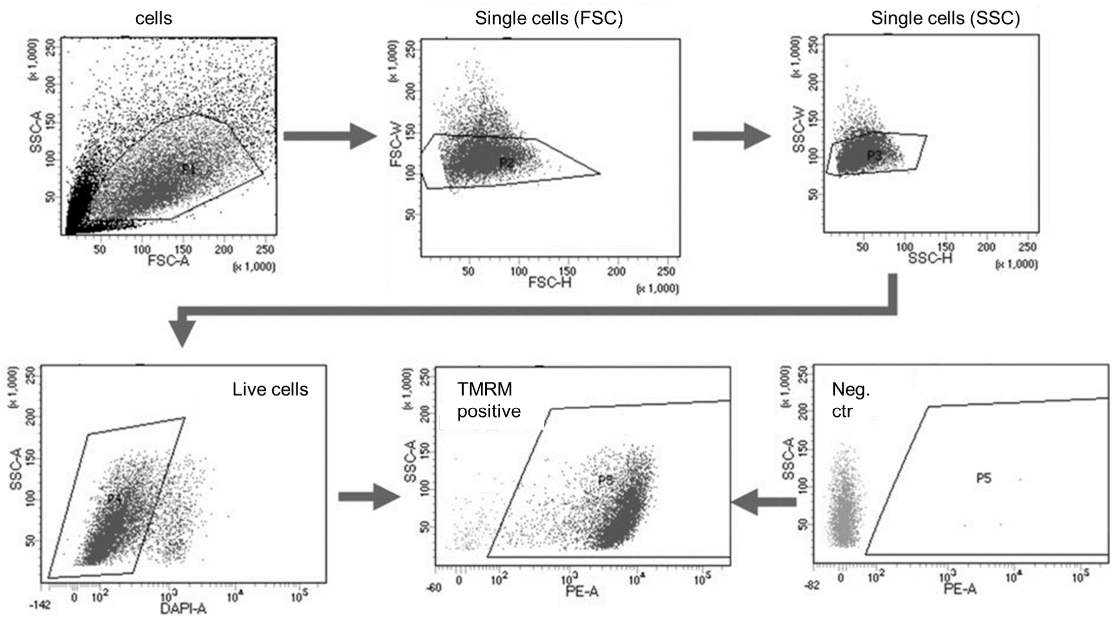

Supplementary Figure 1. Gating strategy of hiPSC-CMs stained with TMRM.

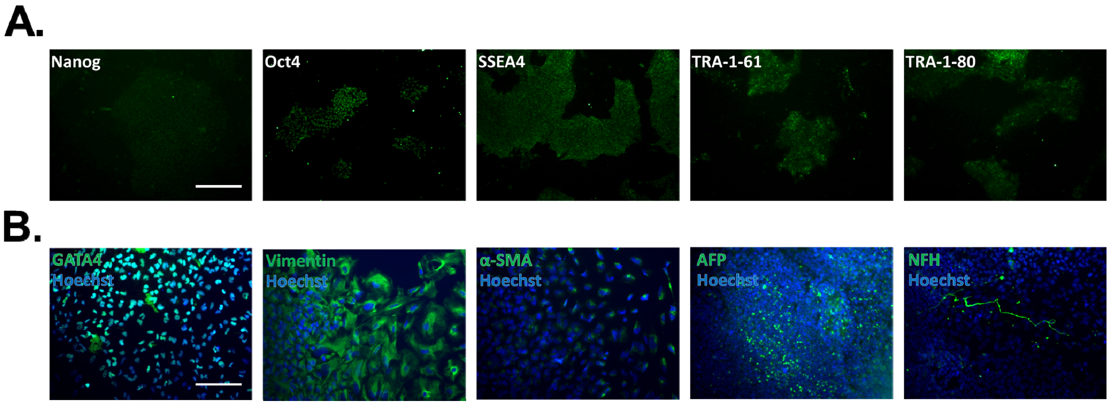

Supplementary Figure 2. Characterization of hiPSC.1. **(A.)** Immunofluorescence analysis of pluripotency markers (NANOG, OCT4, SSEA4, TRA-1-61, and TRA-1-80). **(B.)** Immunofluorescence analysis of markers of three germ-layers of spontaneously differentiated embryoid bodies (GATA4, Vimentin, α-smooth muscle actin, alpha-fetoprotein, and neurofilament heavy chain. Bar indicates 100 μm.

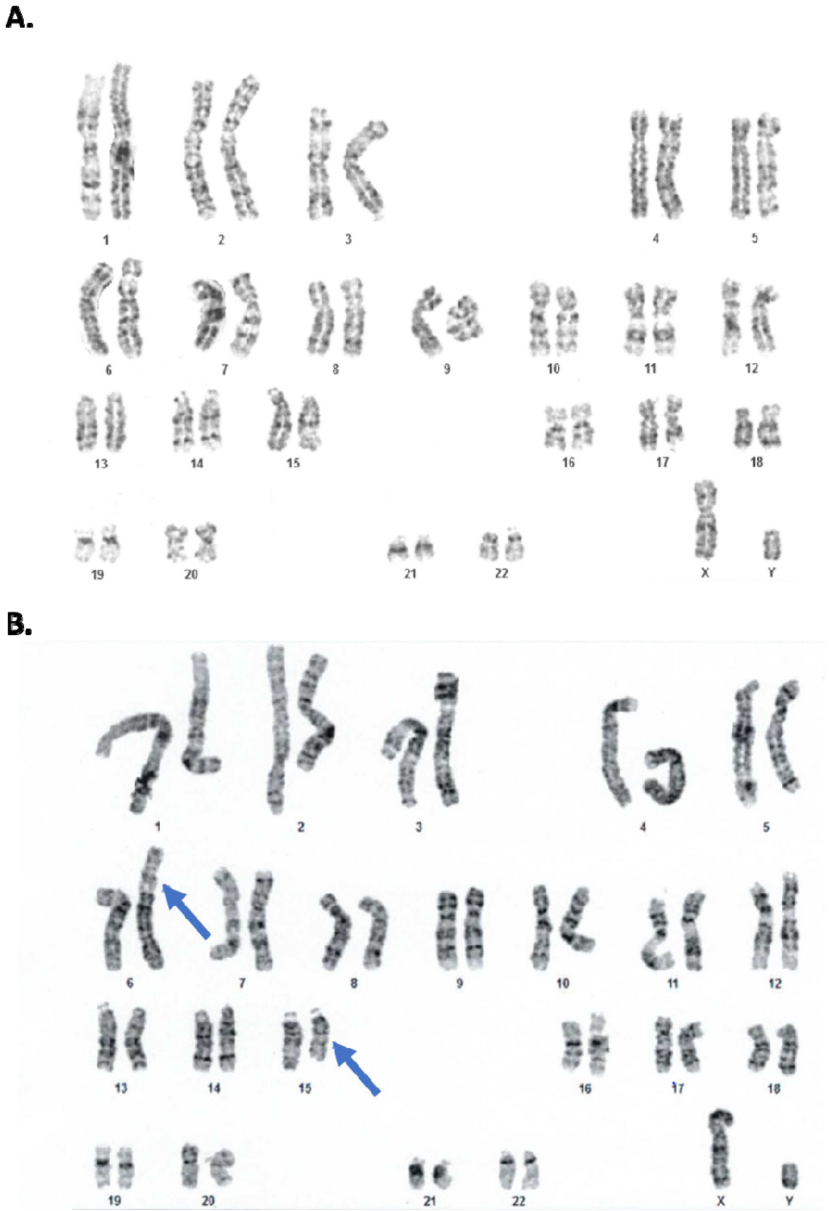

Supplementary Figure 3. Karyotype analysis of (A) hiPSC.1 and (B.) hiPSC.2. Arrows indicating t(6;15)(p21.2;q15) reciprocal translocation.

Karyotype analysis was carried out by Kariogen laboratory (Krakow, Poland). hiPSC.1 derived in our laboratory from BJ fibroblasts was characterized by a proper male set of chromosomes (A.). hiPSC.2 purchased from hipsci.org had a reciprocal translocation t(6;15)(p21.2;q15), indicated by arrows (B.). Identified mutation in hiPSC.2 line did not influence the ability to differentiate to cardiomyocytes, however as soon as was discovered it, the cell line was withdrawn from further experiments. The findings made using mutated cell line were later confirmed with other, non-mutated cell lines. hiPSC.3 was also characterized by the proper set of male chromosomes, as described in other study (Martyniak et al. *in revision*).

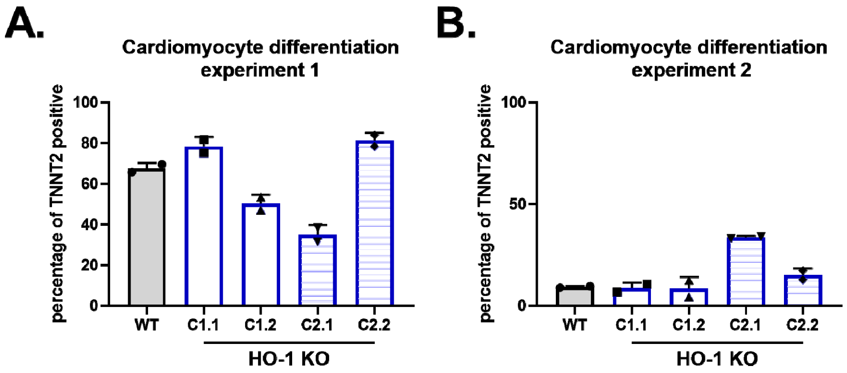

Supplementary Figure 4. Flow cytometric analysis of cardiac differentiation efficiency of WT and HO-1 KO hiPSC.1, based on TNNT2 expression. (A.) Frist differentiation, (B.) Second differentiation. Two replicates in each differentiation.

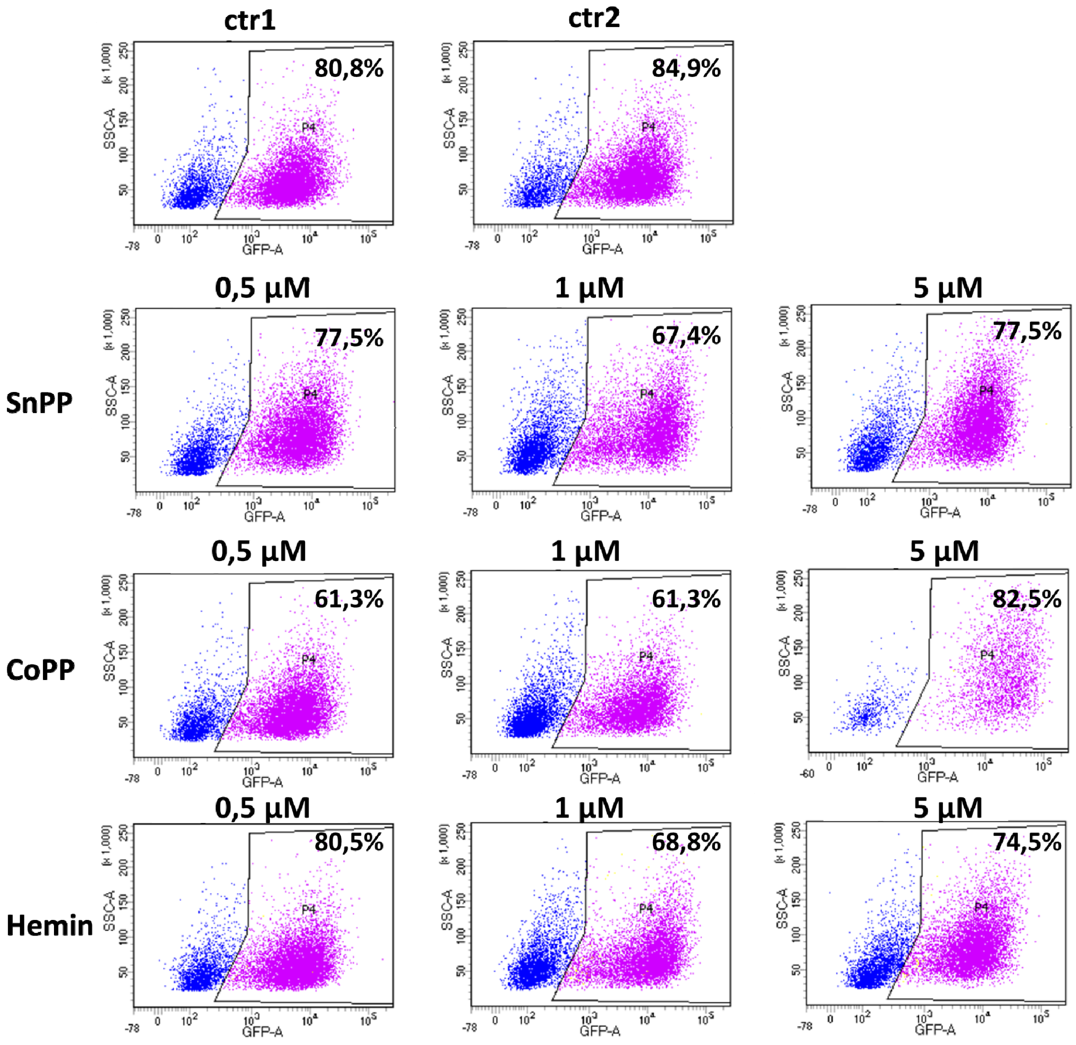

Supplementary Figure 5. Differentiation efficiency. Representative flow cytometry images.

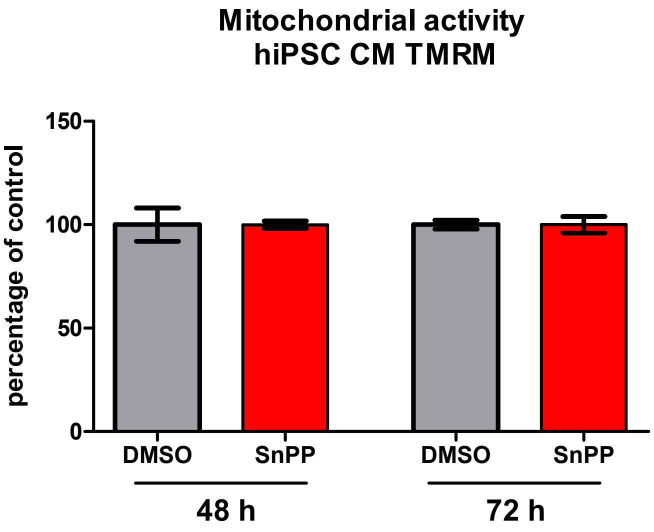

Supplementary Figure 6. Tetramethylrhodamine (TMRM) assay of hiPSC-CMs treated with SnPP. Bars represent median ± SD of N=1 experiment (2 replicates).

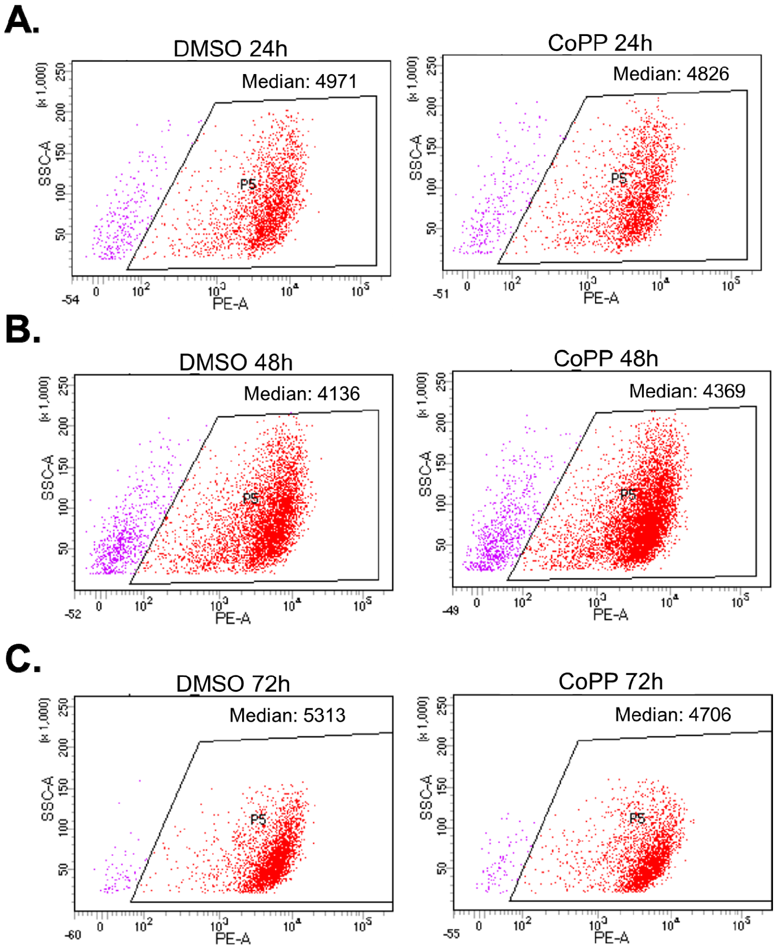

Supplementary Figure 7. TMRM assay of hiPSC.3-CMs. Representative flow cytometry images.

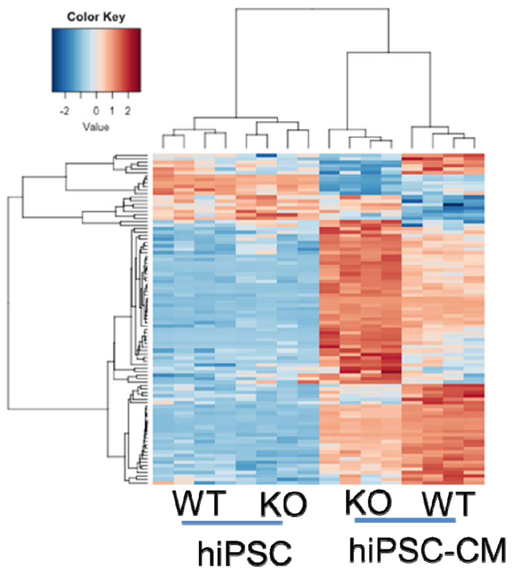

Supplementary Figure 8. Transcriptomic analysis of undifferentiated WT and HO-1 KO hiPSCs and hiPSC-CMs. Hierarchical clustering of differentially expressed genes in undifferentiated (left side) and differentiated cells (right side).

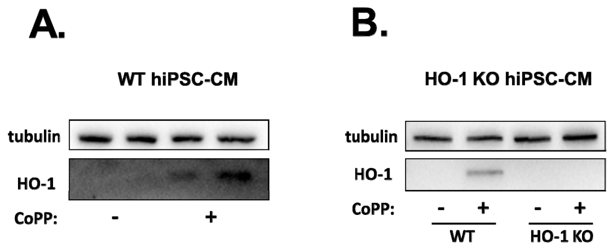

Supplementary Figure 9. Western blot: confirmation of HO-1 induction (or lack of) by CoPP on the protein level in (A.) WT hiPSC.2-CMs and (B.) HO-1 KO hiPSC.2-CMs.
